# Projected climate change effects on suitable habitat for *Pseudosuccinea columella* and *Radix natalensis* in uMgungundlovu, South Africa

**DOI:** 10.1371/journal.pone.0351465

**Published:** 2026-07-16

**Authors:** Mpumelelo Ian Hadebe, Tawanda Manyangadze, Chester Kalinda, Moses John Chimbari

**Affiliations:** 1 College of Health Sciences, School of Medicine, Department of Public Health and Nursing, University of KwaZulu-Natal, Durban, South Africa; 2 Department of Geosciences, Faculty of Science and Engineering, School of Geosciences, Disaster and Development, Bindura University of Science Education, Bindura, Zimbabwe; 3 School of Global Health, University of Global Health Equity (UGHE), Bill and Joyce Cummings Institute of Global Health, Kigali, Rwanda; 4 Durban University of Technology, Dean’s Office, Faculty of Health Sciences, Durban, South Africa; Griffith University, AUSTRALIA

## Abstract

**Introduction:**

Climate change impacts oceanic and atmospheric circulation patterns, precipitation, air and sea surface temperatures leading to altered intermediate host vector distribution, reproduction, and maturation. With temperatures predicted to rise by 1.5°C or more, understanding the influence of environmental characteristics and climatic conditions on distribution of intermediate host vectors, occurrence and habitat suitability is vital in vector borne diseases and their control policies. Hence, the study focused on the predicted changes in habitat suitability of *Pseudosuccinea columella* and *Radix natalensis* in uMgungundlovu district under climate change scenarios.

**Methods:**

Freshwater *Pseudosuccinea columella* and *Radix natalensis* were collected from seven localities in uMgungundlovu using stratified random cluster sampling. Snail samples were collected at 45 sites. The sites were selected based on local knowledge and accessibility. The ensemble model assessed climate variability, while Representative Concentration Pathways (RCP) predicted habitat appropriateness for *P. columella* and *R. natalensis*. Maps were created using the MaxEnt model, and the model’s performance was assessed using the Area Under the Curve (AUC) of the receiver operating characteristic (ROC) curve.

**Results:**

Our study indicates that *P. columella* and *R. natalensis* under current climatic conditions have suitable habitats in the Mpofana, Impendle, Richmond, and Mkhambathini municipalities within the uMgungundlovu district, but the central and northern regions are considered unsuitable. Furthermore, under the RCP4.5 and RCP8.5 climate scenarios, an estimated 22.31% and 22.57% of areas that are presently suitable are expected to become unsuitable for *P. columella* in uMgungundlovu, with fragmented areas and unsuitable areas becoming suitable for *R. natalensis* distribution by 2085.

**Conclusion:**

Climate change could influence the distribution of fasciola intermediate host snails, potentially shifting the hotspots of fascioliasis transmission. Future studies should take into account shifts in land use, evolving agricultural techniques, and the impact of human activities to ensure effective management strategies.

## 1 Introduction

Global warming and climate change are well-established phenomena. Forecasts for the future show changes in rainfall, sea level, oceanic salinity, airflow patterns, and atmospheric and ocean surface temperatures [1]. The changes could impact the rate of development and spread of parasitic organisms that are very susceptible to weather and climate patterns [2]. Changes in climate change could influence parasite reproduction and maturity, potentially altering the distribution and survival rates of intermediate host snails [3]. The World Meteorological Organization (WMO) predicts that the annual global surface temperature from 2023 to 2027 is expected to exceed 1.5 °C above pre-industrial levels in at least one year, influenced by increasing concentrations of heat-trapping greenhouse gases [4]

The connection between climate change and parasitic diseases is highlighted in numerous studies, with some suggesting a rapidly expanding geographical distribution or local prevalence and intensities [5,6]. Climate plays a key role in determining the geographical spread of infectious diseases, whereas weather conditions affect the frequency, intensity, and timing of disease outbreaks [7]. Consequently, extreme weather events such as floods and droughts can influence the occurrence and intensity of disease outbreaks, while long-term shifts in climatic conditions may reshape the geographic distribution and ecological range of infectious diseases [3].

The impacts of climate change contribute to shifts in the geographic spread and occurrence of various diseases, alterations in air quality, and challenges to food security [8]. Average annual air temperatures in South Africa’s inland areas are expected to increase by approximately 3°C to 3.5°C, whereas coastal regions are projected to experience a rise of 1.5°C to 2.5°C. Furthermore, winter rainfall areas will experience a 20–40 mm per decade reduction in their mean annual rainfall, while summer rainfall areas will experience a 40–80 mm per decade increase [9]. The Western Cape and the southern parts of the Eastern Cape, regions characterized by their winter rainfall patterns, are expected to see higher temperatures and less precipitation during the winter season, the summer rainfall regions of Kwa-Zulu Natal, Mpumalanga, and Limpopo are expected to see higher temperatures and more precipitation during the summer and fall [10]

Among the parasitic disease likely to be impact by climate change is Fascioliasis, a significantly neglected zoonotic foodborne disease that poses a considerable threat to human and animal health. Fascioliasis is commonly found in tropical and subtropical areas [11]. Managing Fascioliasis’s risks requires a thorough grasp of its life cycles, host uniqueness, and geographic dispersion. Freshwater snails have gained significant attention from the scientific community due to their role as intermediate hosts for a number of diseases that affect both humans and animals, including schistosomiasis and fascioliasis [12]. Fascioliasis results from an infection caused by trematode parasites belonging to the Fasciola species, specifically Fasciola hepatica (Linnaeus, 1758) and Fasciola gigantica (Cobbold, 1855; Galba Schrank, 1803). The distribution and presence of intermediate host snails play a crucial role in defining the geographic range of fascioliasis [13].

Advancements in technology, along with enhanced statistical and modelling techniques like species distribution models, have significantly improved the understanding of snail distribution. The Species Distribution Model (SDM) assesses the correlation between environmental factors, climate conditions, and species presence to predict their potential distribution and identify suitable habitats [14]. The maximum entropy (MaxEnt) model, recognized as the most advanced machine learning approach among Species Distribution Models (SDMs), was designed to estimate the probability of a species’ presence based on statistical calculations [15]. Since absence data is typically limited and, when available, tends to be unreliable, the MaxEnt model is extensively utilized in species distribution modelling due to its capability to analyze presence-only data effectively [16].

The correlation between climate change and snail-borne diseases, including fascioliasis, has been thoroughly investigated in West Africa, North Africa, and South America [3,5,6], evidence from Southern Africa is limited, especially at sub-national level. Most past modelling studies have been performed at continental or national levels [8,17,18], this may neglect significant local-scale changes in temperature and habitat that influence disease risk. In South Africa, particularly in KwaZulu-Natal, projected increases in temperature and rainfall create conditions that may enhance snail survival and fascioliasis transmission [8,9]. However, few studies have been conducted to evaluate the effects of these climate changes on intermediate host snail distribution at the district level. In order to address this gap, this study applied the MaxEnt species distribution model [14,19], to predict the current and future habitat suitability of *P. columella* and *R. natalensis* in the seven municipalities of the uMgungundlovu district. This study focuses on a local scale. It offers new insights to guide specific control strategies for fascioliasis in both humans and animals.

## 2 Materials and methods

### 2.1 Study area

**Fig 1:**
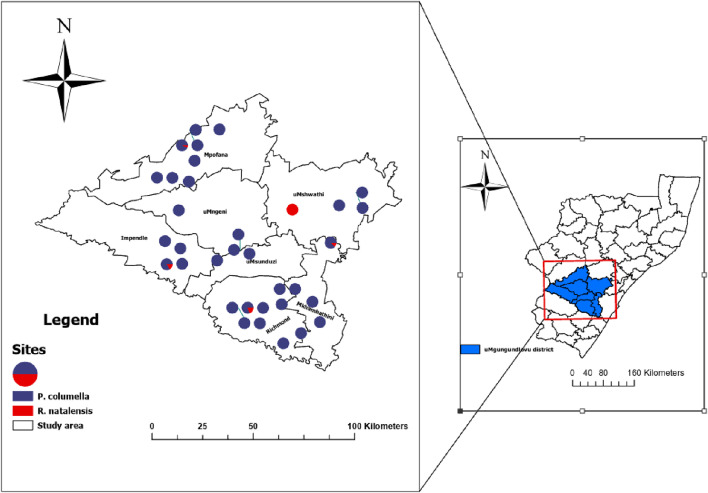
The distribution of *P. columella* and *R. natalensis* across seven municipalities of the uMgungundlovu district. (Map image is the intellectual property of Esri and is used herein under license. Copyright © 2026 Esri and its licensors. All rights reserved) https://dataportal-mdb-sa.opendata.arcgis.com/maps/27bbdd5b041b4ba6b5707dfed5aa3923.

The uMgungundlovu District Municipality is situated in the midland region of KwaZulu-Natal (Fig 1). The uMgungundlovu District Municipality is composed of seven local municipalities and shares its borders with several administrative regions. To the east, it is adjacent to the Ilembe District Municipality, while the Umzinyathi District Municipality lies to the northeast. The Ethekwini Metropolitan Municipality borders it to the southeast, and the Harry Gwala District Municipality is situated to the southwest. To the north, the district is bordered by both the Okhahlamba-Drakensberg World Heritage Site and the Uthukela District Municipality. According to the 2016 Community Survey, uMgungundlovu District Municipality has a population of approximately 1.1 million people, with nearly 60% residing in urban areas. uMgungundlovu District Municipality encompasses an area of roughly 9,600 km^2^, and receives rainfall primarily during the early to mid-summer season (October to February). The northern parts of the district receive an average annual rainfall of approximately 600–800 mm, while the western mountainous regions experience precipitation levels exceeding 1000 mm per year. Furthermore, the district retains a competitive advantage in the realms of agriculture and commercial farming. The existence of six principal rivers and five substantial structures, in conjunction with abundant water resources, establishes uMgungundlovu as one of the top areas in the country for agricultural and livestock produce. Therefore, the significant number of livestock farming within the uMgungundlovu district made it a suitable study area for our research.

#### 2.1.1 Species occurrence data.

A malacological survey was conducted in 7 local municipalities of uMgungundlovu district from the 15–26 April 2024. The sampling sites were selected based on accessibility, knowledge from local people and Google Maps. Sampling was done once at each site for 30 minutes by two team members. The geographical coordinates of each site were recorded using a global positioning system. *Pseudosuccinea columella* was found in 34 sites out of 45 (n = 569) and *R. natalensis* was found in 5 sites out of 45 (n = 16). Only 4 sites had co-occurrence of the fasciola intermediate host snails (Fig 1).

### 2.2 Snail sampling

Two individuals conducted scooping and handpicking snails for 30 minutes at each site as described by [20] meanwhile, an individual documented the geographic coordinates of each location using a handheld GPS device. Snail sampling was conducted at 45 sites within the seven municipalities (Fig 1). The sampled sites for each local municipality were as follows: Impendle [5], Richmond [11], uMsunduzi [3], uMshwathi [5], Mpofana [9], Mkhambathini [8] and uMngeni [4]. The snails collected from each site were counted and identified to the species level based on their morphological characteristics using the shell identification key developed by [21] and subsequently returned to their natural habitats.

#### 2.2.1 Ethics approval.

The University of KwaZulu-Natal Animal Research committee (AREC) issued the ethical approval (Ref no: (AREC/00005912/2023))

### 2.3 Environmental variables

The AFRICLIM version 3 database provided 14 distinct bioclimatic variables, all at a spatial resolution of 30 arc-seconds, which is approximately equivalent to 923 meters. Ten general circulation models (GCMs), were used in the AFRICLIM ensemble model: CNRM-CERFACS-CNRM-CM5, ICHEC-EC-EARTH, NOAA-GFDL-GFDL-ESM2M, CSIRO-QCCCE-CSIRO-Mk3-6-0, CCCma-CanESM2, MPI-M-MPI-ESM-LR, IPSL-IPSL-CM5A-MR, MIROC-MIROC5, MOHC-HadGEM2-ES, and NCC-NorESM1-M. The number of regional climate models (RCMs) was later lowered to five: CCCma-CanRCM4_r2, CLMcom-CCLM4-8-17_v1 (4 GCMs), DMI-HIRHAM5_v2, KNMI-RACMO22T_v1 (2 GCMs), and SMHI-RCA4_v1 (10 GCMs), in conjunction with the WorldClim current baseline, with two representative IPCC-AR5 concentration scenarios (RCP4.5 and RCP8.5) evaluated.

This model of the ensemble was employed due to its ability to deliver robust evaluations of anticipated climate variability while effectively minimizing bias [22]. Representative Concentration Pathways (RCPs) were employed to forecast the future distribution of suitable habitats for intermediate host snails within the uMgungundlovu District. These pathways outline four potential future climate scenarios based on projected greenhouse gas emissions by 2085. RCP 2.6 represents a low-emission scenario, while RCP 4.5 and RCP 6.0 illustrate intermediate levels of greenhouse gas emissions [23]. In contrast, RCP 8.5 corresponds to a scenario characterized by extremely high emissions [24]. Without additional mitigation measures, emissions are expected to follow a path fluctuating between RCP 6.0 and RCP 8.5. In contrast, RCP 2.6 represents an ideal scenario where all emission reduction strategies are effectively implemented, keeping global temperature rise below 2°C compared to pre-industrial levels. Given the near impossibility of achieving RCP 2.6, this study utilized RCP 4.5 and the high-emission scenario of RCP 8.5 to project changes in the geographic distribution of suitable habitats for Fasciola intermediate host snails.

### 2.4 Modelling and evaluation

MaxEnt models were run using default feature classes and regularization settings. Default MaxEnt settings were used as they are widely validated, reduce risks of overfitting, and are appropriate for modelling broad-scale climatic suitability. Variable importance was evaluated through two measures. First, during each iteration of the training algorithm, the increase in regularized gain was assigned to the corresponding variable (or reduced if the absolute value of lambda decreased). Second, permutation importance was assessed by randomly permuting values of each predictor among the training and background data, and recording the resulting drop in training AUC, normalized to percentages. These complementary measures, analogous to a jackknife test, provided insight into predictor influence, though results must be interpreted cautiously when predictors are correlated.

MaxEnt version 3.3.3k (https://www.cs.princeton.edu/~schapire/maxent) was employed to develop habitat suitability maps for both *P. columella* and *R. natalensis*. For each species, the presence data was randomly split so that 75% served as the training dataset while the remaining 25% was reserved for testing the model’s predictive performance [25,26]. To assess the relative influence of environmental factors on the MaxEnt model, a variable contribution analysis was conducted. The explanatory power of each environmental variable was evaluated using MaxEnt’s jackknife method, providing a heuristic assessment of their individual contributions to the model [27]. This analysis illustrated the model’s predictive performance across different training scenarios—whether by excluding individual variables, considering each variable in isolation, or using all variables together. The effectiveness of the fitted MaxEnt model in differentiating between suitable and unsuitable habitats for *P. columella* and *R. natalensis* was evaluated using the area under the receiver operating characteristic (ROC) curve (AUC). AUC scores range from 0 to 1, where a score of 1 represents perfect discrimination, and values of 0.5 or below indicate that the model’s performance is no better than random chance. In practical terms, models with AUC values closer to 1 exhibit superior discriminative ability, and an AUC exceeding 0.75 is generally considered indicative of adequate performance and overall value [28]. Habitat suitability indices for *P. columella* and *R. natalensis* were computed, producing values on a scale from 0 to 1. A score of 0 indicates an environment that is unsuitable for the species, while higher scores denote increasingly favorable conditions for their occurrence [29].

## 3 Results

The three variables that contributed most significantly to forecasting optimal environments for the spatial distribution of *P. columella* were, rainfall in the wettest quarter (30.9%), potential evapotranspiration moisture (26%), and annual temperature range (23.4%), among the 13 bioclimatic variables employed. In contrast, among the three bioclimatic variables in the model, the two that most significantly contributed to predicting suitable habitats for *R. natalensis* were rainfall during the wettest quarter (70.7%) and maximum temperature warmest month (27.6%) (Table 1).

**Table 1:** Bioclimatic variables incorporated into the MaxEnt model to predict the future suitable habitats for *P. columella* and *R. natalensis*, indicating their percentage contribution to the model’s performance.

Bioclimatic variable	Description	Percentage contribution	
		*P. columella*	*R. natalensis*
Bio1	Mean annual temperature	4.8	–
Bio2	Mean diurnal range in temperature	0.1	–
Bio3	Isothermality	1.6	–
Bio4	Temperature seasonality	0.4	–
Bio5	Max temp warmest month	3.5	27.6
Bio6	Min temp coolest month	1.1	–
Bio7	Annual temp range	23.4	–
Bio13	Rainfall wettest month	1.4	–
Bio14	Rainfall driest month	0.2	–
Bio16	Rainfall wettest quarter	30.9	70.7
Pet	Potential	26	–
Mimq	Moisture index moist quarter	6.2	–
Miaq	Moisture index arid quarter	0.4	1.7

**Fig 2:**
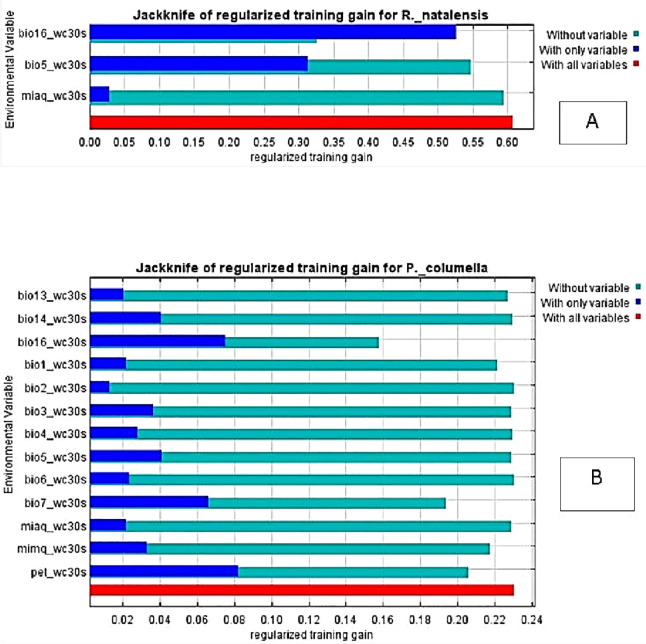
Evaluation of the environmental variables’ contribution in the MaxEnt model for *R. natalensis* (A) and *P. columella* (B) using Jack-Knife plots.

The environmental variable that produces the greatest regularized training gain when used alone to model the habitat suitability of *P. columella* was the Potential evapotranspiration moisture variable (pet). Environmental variable exhibiting the highest regularized training gain when modelling the habitat suitability of *R. natalensis* in isolation was rainfall during the wettest quarter (Bio16) (Fig 2). For both *P. columella* and *R. natalensis*, rainfall wettest quarter (Bio16) is the environmental variable whose exclusion results in the most substantial reduction in model gain.

**Fig 3:**
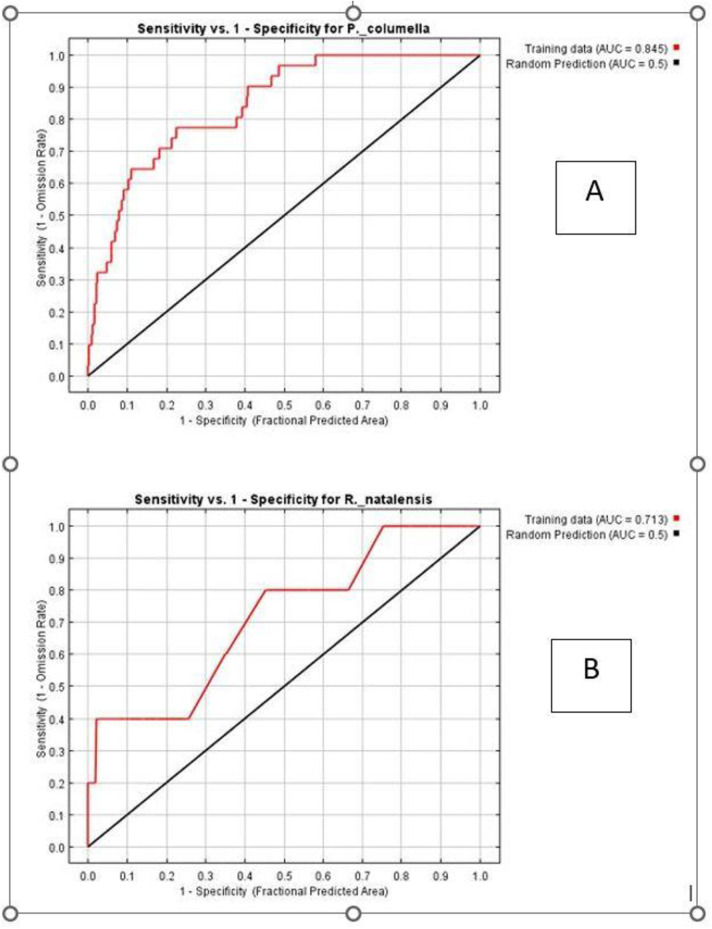
The outcomes of the AUC of the ROC when developing the model of habitat suitability for *R. natalensis* (B) and *P. columella* (A).

The percentage contribution of bioclimatic factors to the MaxEnt model’s performance was used to estimate the existing and forecast future appropriate habitats for *P. columella* and *R. natalensis*. For *P. columella* and *R. natalensis*, AUC of the ROC were 0.845 and 0.713, respectively (Fig 3).

### 3.1 Habitats suitable for *P. columella* and *R. natalensis* under current climatic scenario

**Fig 4:**
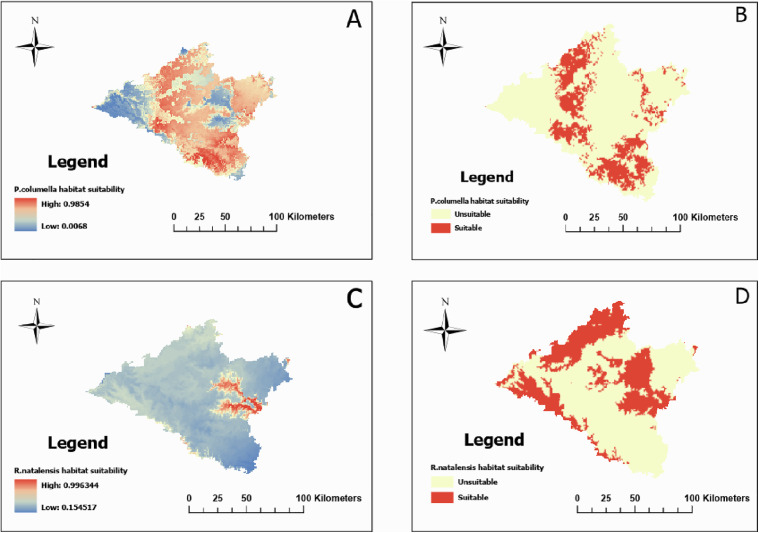
**(A)** Threshold and **(B)** *P. columella* habitat suitability. **(C)** Threshold and (D) the habitat suitability of *R. natalensis* under present climatic conditions in the uMgungundlovu District, KwaZulu-Natal, South Africa. (Map image is the intellectual property of Esri and is used herein under license. Copyright © 2026 Esri and its licensors. All rights reserved).

The habitat suitability for *P. columella* and *R. natalensis* in the uMgungundlovu district under current climate conditions is shown in Table 2 and Fig 4. In uMgungundlovu, a total area of 2152.78 km^2^ is suitable habitat for *P. columella*, comprising 22.42% of the total municipality landmass, while 3388.88 km^2^ (35.27%) total municipality landmass is suitable for *R. natalensis*. Appropriate regions for the distribution of *P. columella* are located within the Mpofana municipality, extending as a corridor across the uMgeni municipality towards the southern section of the Impendle municipality. The currently suitable habitats are dispersed between the Richmond and Mkhambathini municipalities, with further habitats located in the northern region of the uMshwathi municipality. The centre region of the uMgungundlovu district (uMsunduzi municipality) currently represents an unsuitable habitat for the distribution of *P. columella*, as is the northern section of the Impendle municipality. The external margins of Mpofana, Impendle, and Richmond currently constitute suitable habitats for *R. natalensis*. Moreover, uMshwathi municipality is currently a suitable region for *R. natalensis*. The southern region of Richmond and Mkhambathini encompasses the largest area of currently unsuitable habitats. The “maximum training sensitivity plus specificity” criterion used to differentiate between “suitable” and “unsuitable” habitats achieved optimal results as advised by [30].

**Table 2:** Habitat suitability of *P. columella* and *R. natalensis* under prevailing conditions.

	Habitat suitability	*P. columella*		*R. natalensis*	
		Area(km^2^)	%	Area (Km^2^)	%
Current conditions	Suitable	2152.78	22.42%	3388.88	35.27%
	Not suitable	7449.58	77.58%	6220.50	64.73%
		**9602.36**	100	**9609.38**	100

### 3.2 Suitable habitats for *P. columella* and *R. natalensis* under future climate scenario

**Fig 5:**
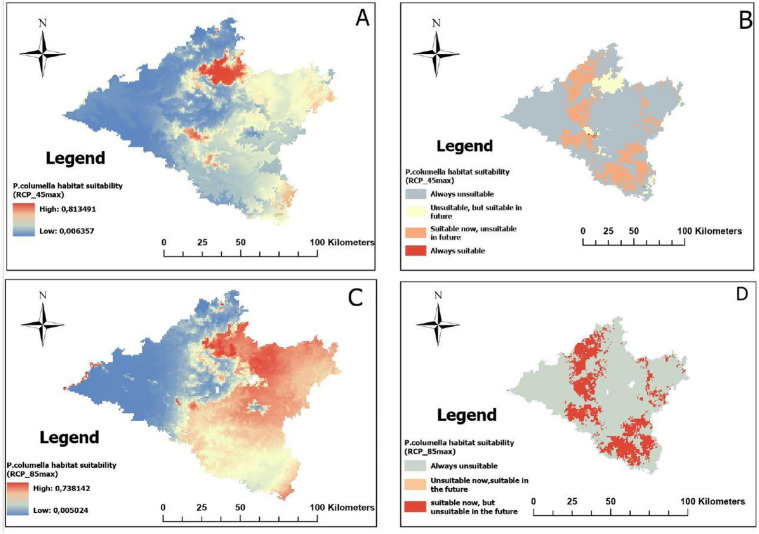
Habitat suitability of *P. columella* in the context of future climatic scenarios (A)threshold RCP4.5 and (B) RCP4.5 scenarios. (C) threshold at RCP8.5max and RCP8.5max in the uMgungundlovu area of KwaZulu-Natal. (Map image is the intellectual property of Esri and is used herein under license. Copyright © 2026 Esri and its licensors. All rights reserved).

**Fig 6:**
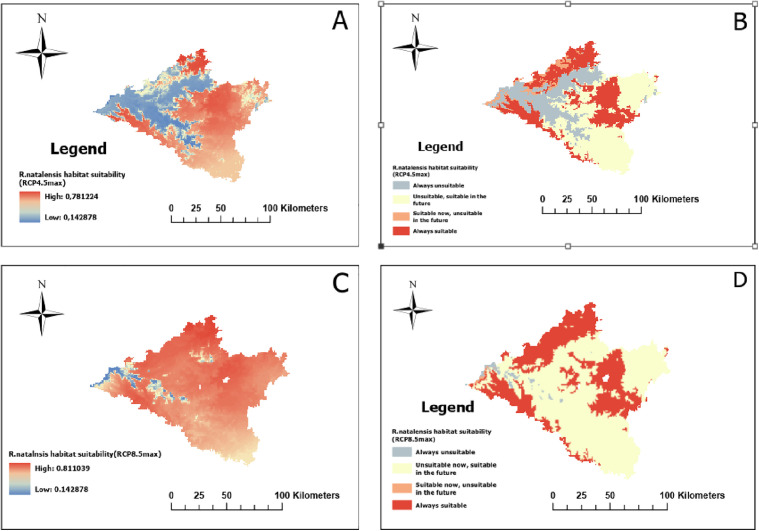
Future habitat suitability of *R. natalensis* under projected climate scenarios: (A) threshold at RCP4.5max and (B) RCP 4.5max. (C) threshold at RCP 8.5max and **(D)** RCP 8.5max in the uMgungundlovu district, KwaZulu-Natal. (Map image is the intellectual property of Esri and is used herein under license. Copyright © 2026 Esri and its licensors. All rights reserved).

Fig 5 illustrates the predicted habitat suitability for *P. columella* under both the RCP4.5 and RCP8.5 climate scenarios for the year 2085. The projections reveal a substantial divergence between the habitats currently considered suitable and those expected in 2085 under both scenarios. Under RCP4.5, there is a marked reduction in suitable habitats across all municipalities within uMgungundlovu, with fragmented zones appearing between the Impendle and uMgeni municipalities, in the northern section of Mpofana municipality, and in the southern part of Mkhambathini municipality. In contrast, the RCP8.5 scenario predicts a complete loss of always most suitable habitats. About 30.92% and 34.68% of currently appropriate areas will remain suitable under the RCP4.5 and RCP8.5 climate scenarios, respectively, while 42.83% and 63.71% of currently unsuitable habitats will become suitable for the distribution of *R. natalensis* in RCP4.5 and RCP8.5 (Table 3). By 2085, under the RCP4.5 and RCP8.5 climatic scenarios, there will be a contraction, shift, and decline in the habitat suitability of *P. columella*, as regions previously deemed suitable, such as iMpofana, uMshwathi, Richmond, and uMkhambathini municipalities, may become unsuitable. Conversely, under high greenhouse gas emissions (RCP8.5), certain areas of Mpofana and Impendle municipalities are expected to become suitable for the proliferation of *R. natalensis* (Fig 6). Furthermore, under the RCP4.5 and RCP8.5 climate scenarios, an estimated 22.31% and 22.57% of areas that are presently suitable are expected to become unsuitable, whereas 4.98% and 0.04% of habitats currently deemed unsuitable may transition into suitable environments for *P. columella* distribution under the RCP4.5 and RCP8.5 scenarios, respectively (Table 3).

**Table 3:** Changes in *P. columella* and *R. natalensis* habitat suitability under RCP4.5 AND RCP8.5 climatic scenarios in 2085.

Habitat suitability	Possible changes	*P. columella*				*R. natalensis*			
		RCP 4.5		RCP 8.5		RCP 4.5		RCP 8.5	
		km^2^	%	km^2^	%	km^2^	%	Km^2^	%
Not suitable	Always unsuitable	6967.33	72.57	7332.65	77.39	2109.53	21.96	126.53	1.33
	Currently unsuitable but suitable in the future	478.31	4.98	4.0	0.04	4114.40	42.83	6043.09	63.71
Suitable	Currently suitable but unsuitable in the future	2141.91	22.31	2138.65	22.57	412.29	4.29	25.86	0.27
	Always most suitable	13.42	0.14	0	0	2969.74	30.92	3289.69	34.68

**Fig 7:**
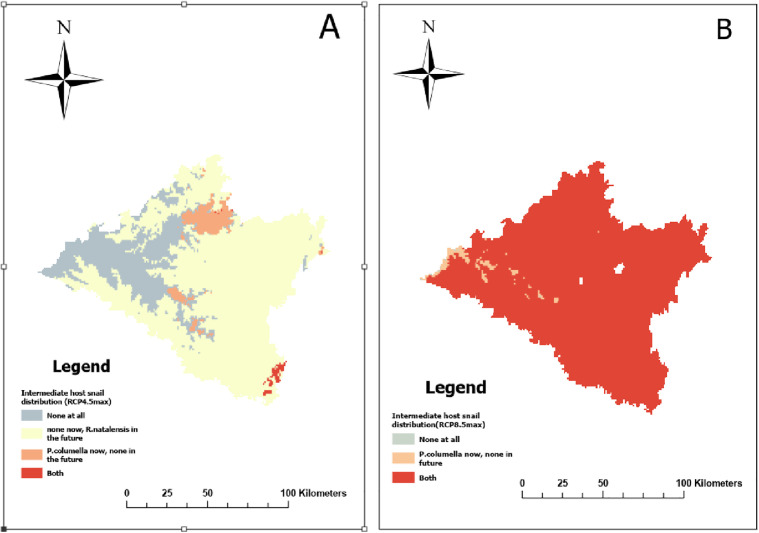
Possible future suitable and unsuitable habitats for *P. columella* and *R. natalensis* under (A) RCP_4.5 scenario and B) RCP 8.5 scenario in the uMgungundlovu district. (Map image is the intellectual property of Esri and is used herein under license. Copyright © 2026 Esri and its licensors. All rights reserved).

The potential suitable habitats for the co-occurrence of the Fasciola intermediate host snails *P. columella* and *R. natalensis* are illustrated in Fig 7. In the RCP4.5 climatic scenario (A), it is evident that only 0.65% of the area is predicted to be suitable (Red) for the distribution of both fasciola intermediate host snails. Conversely, under the RCP8.5 climatic scenario, it is predicted that 98.34% habitats will be suitable for the co-occurrence of the Fasciola intermediate host snails.

The predicted distribution patterns of *P. columella* and *R. natalensis* under the RCP4.5 and RCP8.5 scenarios are illustrated in Table 4. Predictions indicate that around 0.65% of habitats will consistently be optimal for the co-occurrence of *P. columella* and *R. natalensis* under the RCP 4.5 scenario, while 98.34% will be suitable under the RCP 8.5 scenario, this is anincrease of suitable habitat area from 62.25 km^2^ (RCP 45) to 9413.14 km^2^ (RCP 85).

**Table 4:** Changes in habitats suitable for the co-occurrence of *P. columella* and *R. natalensis* in the uMgungundlovu district.

Habitat suitability	Possible changes	RCP_4.5max		RCP_8.5max	
		Area (km^2^)	(%)	Area (km^2^)	(%)
Not suitable	None	435.10	4.53	6.0	0.06
	None now, *R. natalensis* in the future	7027.68	73.15	0	0
Suitable	*P. columella* now, but none in future	2082.18	21.67	153	1.60
	Both present	62.25	**0.65**	9413.14	**98.34**

## 4 Discussion

The MaxEnt model was employed to evaluate and forecast the current and future distributions of suitable habitats for *P. columella* and *R. natalensis* under two climate scenarios (RCP4.5 and RCP8.5) for the year 2085. Despite the use of limited datasets, the model effectively predicts snail distribution [31]. The ROC analysis produced high AUC scores for predicting suitable habitats, 0.845 for *P. columella* and 0.713 for *R. natalensis*, demonstrating the reliability of the results. Although *R. natalensis’s* AUC score (0.713) is within an acceptable range, it is noticeably lower than *P. columella’s*. AUC is recognized to have limitations because it does not account for calibration and is impacted by geographic range and prevalence [32]. The low performance might be a result of *R. natalensis’s* wider ecological tolerance [33], and dependence on fine-scale habitats that are not resolved by coarse climatic predictors, like irrigation canals and temporary ponds [20]. Therefore, rather than being exact local forecasts, our projections for *R. natalensis* should be considered indicative of broad-scale suitability.

The results show that different bioclimatic variables had an effect on the distribution of *P. columella* and *R. natalensis*, with *P. columella* mainly influenced by rainfall during the wettest quarter, annual temperature range and potential evapotranspiration moisture. This suggests that the two primary variables identified contain the highest level of information not present in the other variables. On the other hand, *R. natalensis* was influenced by rainfall during the wettest month and maximum temperature of the warmest month. These bioclimatic variables play a critical role in the amount of water within a habitat. In Fig 1, the light blue bars (lacking variables) are shorter than the red bars (incorporating all variables), indicating that the model’s predictive ability improves when the relevant factors are utilized. A study carried out by Manyangadze, Chimbari [34] indicated that snail abundance is contingent upon the quantity of water present in an environment. This is because snails require water to survive and thrive [35]. However, snail populations diminish when there is an excessive amount of water particularly during flash floods. Consequently, rainfall contributes to the creation of temporary snail habitats and facilitates the development of new ones [36]. The MaxEnt model demonstrated outstanding predictive accuracy, effectively representing the distribution and habitat suitability of *P. columella* and *R. natalensis*, as indicated by the results, which exceeded 0.75 [19,25,26,37,38]

The temperature responses of *P. columella* and associated lymnaeids generally exhibit optimal fecundity at moderate warm temperatures (mid-20s °C), with a decline at elevated temperatures; invasive populations may demonstrate local adaptation that alters thermal optima, yet the fundamental physiological limitations (reduced egg viability and increased mortality at extreme temperatures) persist [33]. Consequently, in uMgungundlovu, the combined effect of increased rainfall during the wettest month enhancing habitat availability and moderate temperatures promoting fecundity demonstrates the seasonally increased presence of *P. columella*, but excessive heat and flooding events may limit or reset populations. Rainfall during the wettest month is likely responsible for the occurrence of *R. natalensis*. This is due to multiple mechanisms, including moisture-dependent egg and juvenile survival, habitat re-establishment, and scour/washout at high intensity [39]. These processes contribute to shortened hatching times and improved early survival at moderate warm temperatures, while extreme heat or dry spells reduce egg viability [33].

Current suitable habitats for *P. columella* are dispersed across significant number of sites in different municipalities with uMgungundlovu district. The distribution is concentrated in the Mpofana, uMgeni and south portion of Impendle municipality. The habitat suitability *of P. columella* can be further observed within Richmond and uMkhambathini municipality and patchy suitable habitats are visible in the uMshwathi municipality. These patterns may be attributed to the climate in uMgungundlovu district that changes from being warm to hot and humid in the lower southeastern parts due to the warm Indian Ocean influence to a more temperate climate in the northwestern parts where altitude has a stronger influence. Malatji, Lamb [40] stated that the dispersion of the snails is influenced by climatic and ecological factors, including temperature, precipitation, habitat characteristics, and soil type. The habitat suitability of *R. natalensis* also forms across different municipalities with a different pattern than that of *P. columella*. Current habitat suitability for *R. natalensis* forms horizontally across the Mpofana municipality, towards the outer boundary of the Impendle municipality. The pattern continues vertically down on the western boundary of the district. Richmond and uMkhambathini are not suitable habitats for *R.bnatalensis* whilst uMsunduzi is not suitable for both snails. This may be because the majority of regulated industrial processes are situated within the Msunduzi municipality [41].

The uMgungundlovu district is expected to experience a shift in annual rainfall and an increase in annual average temperatures from 2021 to 2050, as indicated by the RCP 8.5 scenario. By 2050, the district is expected to experience impacts from elevated annual average temperatures, this will negatively affect food and water security, potentially leading to reduced agricultural yields and increased evaporation rates [42]. According to Dube, Kalinda [33], temperature increases may change the distribution and favourable conditions for intermediate host snail (IHS) fecundity, growth, and survival.

Our study showed that under both the RCP 4.5 and RCP 8.5 climatic scenarios, the always unsuitable habitats increase suggesting that future climatic conditions may be too harsh for the survival of *P. columella*. uMgungundlovu’s projections of the district’s annual average number of extreme rainfall days under the RCP 8.5 scenario for the years 2021–2050 indicate that an increase in rainfall days is probably going to lead to more severe storms and flooding incidents throughout the district [43]. A rapid escalation in flow volume and velocity is predicted to lead to elevated water levels and the displacement of IHS from previously sheltered habitats [44]. This may lead to the disappearance of suitable habitats as observed.

Our study further shows that the future habitat suitability for *R. natalensis* under both RCP4.5 and RCP8.5 climatic scenarios. Interestingly, it can be observed in both scenarios that there may be a decrease in unsuitable habitats and habitats that are currently unsuitable are predicted to be suitable, especially areas around the Mpofana, uMgeni and Impendle municipalities. According to Quayle, Appleton [44], water temperature plays a crucial role in influencing snail fecundity, and with the exception of *R. natalensis*, all the species studied appear to be sensitive to temperature changes. *Fasciola* and its hosts may find new habitats as a result of climate change as some areas will experience increased precipitation while others will experience very low temperatures [45].

Under the RCP 8.5 climatic scenario, a small patchy habitat along Impendle, Impofana and uMgeni, was predicted to be suitable habitats for *P. columella* only and the rest of the district was predicted to be suitable for *R. natalensis*. The difference between habitat suitability under 4.5 and 8.5 conditions could be attributed to pollution, land-use and human activities around these snail habitats. According to Mereta, Abaya [46], land-use changes, such as agricultural development and irrigation projects, are closely linked to increased snail habitat availability. Changes in snail habitat suitability over the past 30 years were caused by climate change and land-use changes [47].

Nevertheless, the lack of overlap is also informative, as it indicates the ecological niche division possibly related to different tolerance levels to *R. natalensis* dominance in irrigation and ephemeral pools and *P. columella* in more permanently, and typically, stagnant waters [20]. Including this perspective of co-occurrence further enhances the interpretation of such habitat suitability analysis via integration of single species distribution models with community level interactions, imperative for assessing climate change driven transmission hotspots.

*Pseudosuccinea columella* and *R. natalensis* have co-existing suitable habitats only in the Richmond and uMkhambathini municipalities, with the rest of the municipalities that are currently not suitable for the distribution of *R. natalensis* being potentially suitable habitats in the future. Our results are consistent with those of Malatji, Lamb [40] who suggested that *P. columella* is capable of coexisting with *R. natalensis* in certain regions of South Africa. The co-occurrence analysis in Fig 7 highlights the possible interactions between *P. columella* and *R. natalensis*. Overlapping regions indicate where the species are exposed to the same climatic and environmental conditions and could, therefore, facilitate the trematode transmission cycle together [48].

Previous studies highlight that climatic shifts can expand or contract suitable habitats for freshwater snails depending on the model and scenario [49]. These findings are consistent with our findings. Furthermore, reviews and recent studies for *P. columella* suggest ongoing distribution expansion and ecological plasticity [50,51]. However, a limited number of studies directly compare various GCMs, downscaling methodologies, or incorporate hydrology with climate predictors for these two species in Africa – a deficiency that undermines the generalizability of fine-scale estimates. Consequently, although our findings correspond with the broad patterns documented in other studies (range shifts and sensitivity to temperature/precipitation), they must be interpreted with caution and preferably corroborated by multi-model ensembles and hydrologically informed datasets utilized in regional analyses [49,52]

Our findings highlight the urgent need for active, climate-aware measures to manage fascioliasis risk effectively in KwaZulu-Natal. The expected geographic spread of suitable habitats for *P. columella* and *R. natalensis* requires proactive surveillance and focused snail control strategies. The use of species distribution models, such as MaxEnt, to predict new hotspot areas and focus on active snail and disease monitoring at municipalities with potential risk, like uMgungundlovu, before cases rise. Similar methods have been suggested for climate-related shifts in snail distribution in South Africa, improving public health planning [53]. Implement integrated strategies for controlling snails, such as WASH improvements, aquatic vegetation management, and targeted mollusciding. Work together with the human and animal health sectors to accomplish this. Tanzania and Rwanda are testing a One Health approach to control schistosomiasis and fascioliasis. This demonstrates the advantages of collaboration between sectors [54].

The primary limitation of this study, is the duration of the sampling (April 15 to April 26) which did not cover all seasons, leading to our study producing narrow snapshot. Since the presence and detectability of aquatic snails differ seasonally, short single-season sampling can bias habitat associations and SDM performance [55,56]. According to [57] and [58], seasonal shifts in reproduction an abundance for freshwater snails has been documented; a single late- summer/early-autumn window may miss these dynamics. Furthermore, the use of the AR5 RCP-based forecasts is defensible because the AFRICLIM offers downscaled, bias-corrected projections specific to African contexts, which are still often used in ecological modelling research [22], but is a limitation for this study since there is a more recent concept known as Shared Socioeconomic Pathways (SSPs), which incorporate socioeconomic narratives and emissions trajectories, was established in the Sixth Assessment Report (AR6) [59]. Future research should take into account AR6/SSP scenarios when they become available in regionally downscaled datasets for Africa.

Future studies may need to sample for *Fasciola* IHS in all the seasons to better understand the interaction of these IH with the environment and climatic variables. Brief sample periods may lead to unrepresentative conclusions when detection varies across the season; it is advisable to employ replicate or multi-season designs to reduce this bias [60,61].

Freshwater snail projections on habitat suitability that are informed by climate based SDMs, carry inherent uncertainties arising from the coarse nature of GCM outputs and hydrological complexity at micro-scales [62]. The differences across climate models and internal climate variability can have significant divergence in temperature and precipitation projections, especially at local scale, potentially skewing snail habitat predictions. Snail modelling studies regularly predict climate-induced suitability without considering hydrological factors; for instance, Li [17] highlighted divergent shifting range projections under different SSPs, emphasizing scenario-dependent uncertainty, while Aksu et al. [63], highlighted that the inclusion of hydrological and land-cover variables significantly alters predictions.

Recent work in uMgungundlovu District of KwaZulu-Natal has shown that the invasive *P. columella* is abundant and widespread across both permanent and temporary aquatic habitats, with its abundance influenced significantly by water pH and other physicochemical parameters [64]. This suggests that snail monitoring (both spatially and temporally) linked to environmental assessment is a vital early-warning tool. Programs should include targeted molluscicide application where snail abundance is high, modification of water bodies to reduce stagnation or vegetation which favours snails, and integration of livestock surveillance with local veterinary dipping schemes. Also, work by Nyagura et al. [65], confirms overlapping distributions of *Fasciola hepatica*, *F. gigantica*, and intermediate snail hosts in KwaZulu-Natal, highlighting the need for coordinated human, animal and snail host control efforts in those overlapping zones.

## 5 Conclusion

Climate change has the potential to modify the distribution of Fasciola intermediate host snails by influencing the expansion or contraction of suitable habitats, thereby affecting the transmission dynamics of fascioliasis. This study assesses both the current and projected future distribution of suitable habitats within the uMgungundlovu District, situated in the midlands of KwaZulu-Natal, under different climate change scenarios. To refine predictive accuracy, future research should extend modelling efforts to encompass the entire KwaZulu-Natal province, enabling a more precise identification of suitable habitats across diverse climate scenarios while enhancing the understanding of interactions between intermediate hosts and their environments. Additionally, a comprehensive assessment of the ecological niche of these hosts should incorporate factors such as land use changes, agricultural practices, and human activities. Strengthening education and awareness among veterinary professionals, farmers, and local communities will also be critical for the effective management of fascioliasis in the context of evolving climatic conditions.

## Supporting information

S1 Data Data. (XLSX)
